# For the Cure of Uterine Diseases

**Published:** 1876-11

**Authors:** 


*For the Cure of Uterine Disorders.*—And, while our hand is in—as a matter of curiosity—we will give what claims to be a remedy for the "cure of all womb diseases." It was handed us by a lady, (who was under treatment for a uterine affection,) who had been complimented with the formulæ by a lady who had tested (so she averred) their merits with success after wasting much money, on many physicians, and after paying no less than $50 for the remedy. Here it is. Let Gynæcologists make a note of it and be happy;

RECIPE FOR MAKING THE BITTERS.

R.—Gum Myrrh.......................................... oz. i
Cloves.......................................... oz. i
Nutmeg.......................................... oz. i
Peruvian bark.......................................... oz. ii
Bark of the root dogwood, dried and powdered.... oz. i

Essence Cinnamon.................... ......................oz.i
Calamus, dried and powdered.................................oz.ii

The above to be mixed with half gallon of rum. shake it up once every day for nine or ten days, then you may put into the rum just as much water as rum. Take one teaspoonful of the bitters before each meal. Live on light diet. If the patient is troubled with palpitation of the heart, take one teaspoonfull of sulphur and cream tartar, mixed together. Take pills, if your bowels are constipated.

RECIPE FOR THE WASH.

Take half peck of red oak bark, half peck dogroot bark ; put the barks into a pot with four gallons water, boil the bark three or four hours, until there is about three quarts of the liquor left, then strain the liquor through a thick cloth, then put it into a jug so it will be ready for use whenever needed ; make a strong suds of castile soap, inject into the womb, or birth place, with female syringe several times, after which inject the decoction made with the barks, and then the ointment is to be injected only once. Let every article injected be about milk warm. The hips of the patient should be elevated about four inches whilst being operated on—should lie on her back, if possible, one hour after each operation. If she can't lie on her back, she must lie on her right side. Continue the above two or three times during the day for nine or ten days, until relief is obtained, during which time the patient should not wash her face and hands in cold water.

RECIPE FOR MAKING THE OINTMENT.

Take one gallon bearfoot root, one gallon comfrey-root, wash them well, boil in water about five times, filled up so the strength will all be out of the roots. When it has boiled enough to have one gallon of the liquor, squeeze out these roots and put into the liquor one quart of white lilly root, one teacupful of the buds of balm of gilead, one handfull of healall dried in the leaves, one handfull of featherfew—boil until there is about one quart of the liquor left, strain it all well, and put in three or four pounds or hog's lard into it, stew until all of the water is out of it, then add one ounce of gum myrrh and stir rapidly for about ten minutes, then strain it again through a thick cloth and put it up in glass bottles or small jar, and then it is ready for use.

Selah! G.

